# Breath-holding spells comorbidity with epileptic seizures in children: VEEG and clinical outcomes

**DOI:** 10.1097/MD.0000000000045741

**Published:** 2025-11-07

**Authors:** Bo Chen, Hongjun Fang, Xiaojun Kuang, Zeshu Ning, Lijun Wang

**Affiliations:** a Affiliated Children’s Hospital of Xiangya School of Medicine, Central South University (Hunan Children’s Hospital), Changsha, Hunan, China.

**Keywords:** breath-holding spells, comorbidities, epileptic seizures, no-epileptic seizures, video electroencephalogram

## Abstract

This study aims to explore the electroencephalogram (EEG) and clinical characteristics of breath-holding spells (BHS) comorbidity with epileptic seizures (ES) in children, providing reference for the rational diagnosis and prognosis assessment of such children. We conducted a prospective summary of the clinical data from patients of BHS comorbidity with ES, who were admitted to Hunan Children’s Hospital from April 2017 to November 2023, by analyzing their EEGs and clinical characteristics. There were 7 patients of BHS comorbidity with ES. BHS and ES appeared successively or simultaneously in the course of the disease. There were 8 “slow” patterns and 5 “slow-flat-slow” patterns in all 13 BHS of 7 cases during ictal EEG. The types of ES included tonic-clonic seizures, epileptic spasms, myoclonic, focal seizures, and so on. In the BHS, 5 cases were controlled, and 2 cases were uncontrolled after psychological and behavioral therapy. In the ES, 5 cases were seizures-free, 1 case with myoclonic and epileptic spasms decreased after treatment using valproate and vigabatrin, and 1 case with multiple seizure types was uncontrolled after being treated with multiple antiseizure medications. The onset age of breath-holding attacks in the BHS-only group was earlier than that in the BHS + epilepsy groups. The abnormal rates of head magnetic resonance imaging (MRI) and EEG in the BHS-only group were lower than those in the BHS + epilepsy groups. The BHS-only group had normal interictal EEG, while 5 out of 7 patients in the BHS + epilepsy group had epileptic waves in interictal EEG. The incidence of the BHS + epilepsy group combined with other diseases was higher than that of the BHS-only group. The rate of developmental delay in the BHS + epilepsy group was higher than that in the BHS-only group. Both differences had statistical significance (*P* < .05). BHS combined with ES may exist in children, especially for patients with head MRI abnormalities, EEG abnormalities, and developmental delay. Diagnosis needs to be based on clinical manifestations and VEEG. If new seizure forms appear in BHS patients, a timely EEG examination should be completed to correctly identify the occurrence of ES. If necessary, antiseizure medication should be given.

## 1. Introduction

Breath-holding spells (BHS) are often induced by factors such as anger, excitement, fear, or pain, manifested as sudden and intense crying, followed by holding breath when exhaling, loss of consciousness, head tilted back, stiffness of limbs and trunk, often accompanied by tremors or clonic movements. After 5 to 10 seconds, cyanosis or pallor appears, followed by crying again and recovery of breathing. Severe BHS can be accompanied by urinary incontinence and transient atonicity. BHS is a common benign non-epileptic seizure event in infancy. The peak age is 6 to 18 months, and the incidence rate is 0.1 to 4.6%.^[1]^ The symptoms of BHS are similar to the generalized tonic-clonic seizures (GTCS), a small number of cases are misdiagnosed as epileptic seizures (ES) and receive additional risks from unnecessary antiseizure medication.^[2]^

An ES is a transient occurrence of signs and/ or symptoms due to abnormal, excessive, or synchronous neuronal activity in the brain.^[3]^ Video electroencephalogram (VEEG) is the most important tool for research, diagnosis, and classification of ES. Based on the clinical and ictal EEG characteristics, the ES is classified into multiple types.

BHS is an ancient topic, and there have been numerous literature studies on its clinical manifestations and treatment in the past. However, a very small number of BHS cases can lead to secondary ES,^[4]^ and in clinical practice, we also found cases of BHS co-occurring with ES. Infancy is the peak age period for the occurrence of BHS and ES. When BHS and ES occur in the same child, especially in children with developmental delays and/or obvious abnormalities in head imaging. In order to avoid misdiagnosis and treatment burden, it is extremely important to accurately diagnose BHS and ES. There have been few literature studies on the BHS comorbidity with ES in the past. In order to help pediatric neurologists and electroencephalologists pay attention to and understand the clinical phenomenon of BHS and ES coexisting, and to manage these children reasonably, we summarized cases and combined literature to explain the topic of the coexistence of BHS and ES.

## 2. Materials and methods

### 2.1. Patients

**Figure 1.**
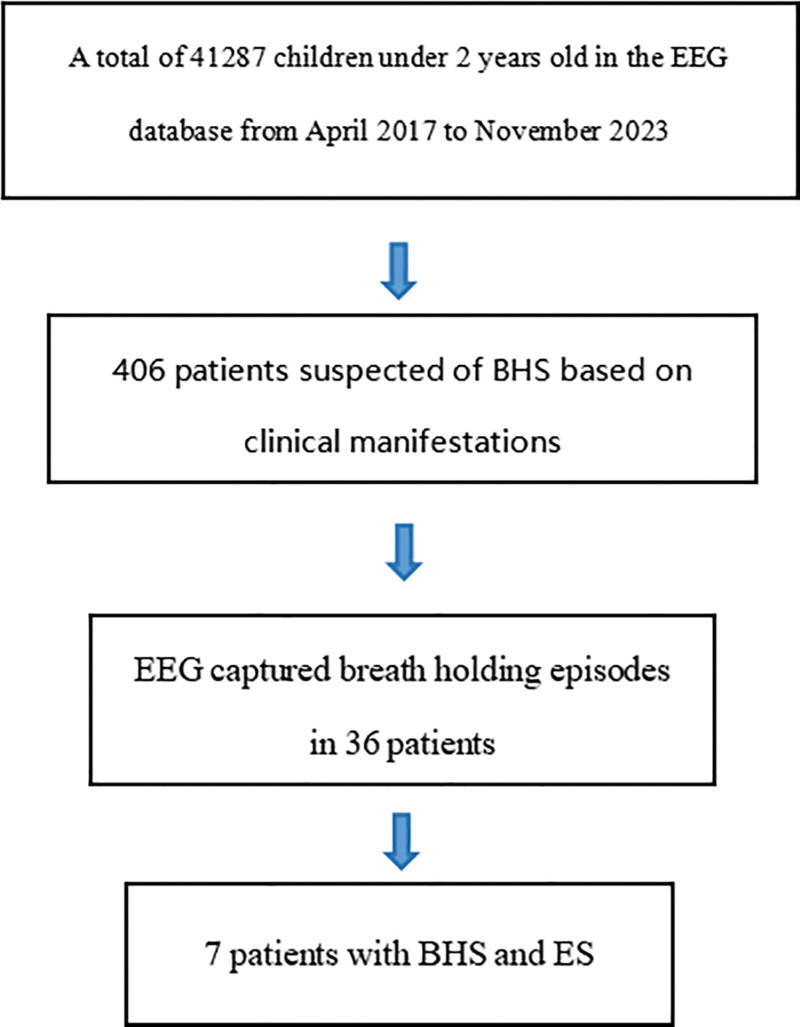
Data collection flow diagram.

We searched the database of the Neurology EEG Monitoring Center of Hunan Children’s Hospital from January 2017 to December 2023 using keywords such as “anoxic seizure” and “breathing holding spells,” and found 36 cases of BHS. Then, we searched for evidence of ES in the VEEG database, disease course, and follow-up records. 7 (19.4%) cases who were detected with BHS and ES using VEEG were found (see Fig. 1). Diagnostic criteria for epilepsy and the seizure type classification are determined according to the ILAE international classification standards.^[5,6]^ The inclusion criteria of BHS were as follows: The sequence often begins with the precipitation by anger, frustration, fear, or injury. The initial precipitating factor is subsequently followed by a violent cry, breath-holding in expiration, cyanosis or pallor, limpness or rigidity, unconsciousness, seizure, relaxation, and recovery without a postictal phase. Postictal confusion is characteristically absent in BHS. A careful physical examination should be performed to exclude other diagnoses prior to confirming the diagnosis.

### 2.2. Methods

All 7 patients completed at least one VEEG while visiting due to BHS or/and ES. VEEG monitoring: We used a 32-channel VEEG monitor (Nihon Kohden EEG-1200C), 19-channel recording electrodes were placed according to the international 10 to 20 system. All EEG connection criteria were met. We conducted electrocardiographic and bilateral upper limb deltoid muscle electromyography recordings. The monitoring time was at least 4 hours. Continuous EEG (cEEG) monitoring: We used the amplitude integrated EEG of 8 channels (Fp1, Fp2, C3, C4, O1, O2, T3, T4) and the original EEG to joint recording.

BHS is a reflexive transient anoxia seizure (TAS), the ictal EEG diagnosis followed patterns of TAS of Gastaut^[7]^: “slow” patterns is generalized voltage gradually increases → gradually decreases, frequency gradually slows → gradually increases δ rhythm. “slow-flat-slow” patterns are generalized voltage gradually increases, frequency gradually slows δ rhythm → generalized low voltage → generalized voltage gradually decreases, frequency gradually increases δ rhythm.

We collected the dates of 7 cases on age, gender, current medical history, past history, family history, physical examination, head magnetic resonance imaging (MRI) (Siemens 3.0T, epilepsy sequence), cardiac ultrasound, blood routine and biochemistry, blood and urine inheritance metabolism screening, development levels and scale, treatment and prognosis of BHS and ES, the results of EEG and clinical follow-up and other dates, and summarized and analyzed them.

### 2.3. Statistical analysis

We implemented the (SPSS 24.0, IBM, Armonk) statistics suited for this analysis. Measurement data with a normal distribution are presented as the mean ± standard deviation, and measurement data with a non-normal distribution are presented as the median. In addition, qualitative data are presented as the number of patients (percentage). The Mann–Whitney *U* test and χ^2^ test were used to compare differences in patient demographics, developmental status, and comorbid conditions between BHS-only and BHS + epilepsy groups.

## 3. Results

**Figure 2.**
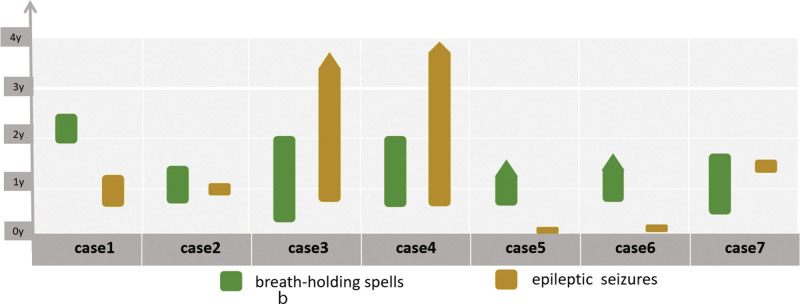
The green and yellow bar charts represent the onset and termination ages of BHS and ES, respectively. The pointed bar chart represents seizures uncontrolled. Cases 2, 3, 4, and 7 exhibited both BHS and ES during a certain period of the disease course. BHS = breath-holding spells, ES = epileptic seizures.

Among the 7 patients, there were 6 males and 1 female. The minimum onset age for BHS was 4 months and the maximum was 1 year and 11 months, while the minimum onset age for ES was 10 days and the maximum was 1 year and 5 months; During the course of the disease, BHS started earlier than ES in 3 cases and ES earlier than BHS in 3 cases, BHS and ES started simultaneously in 1 case, and there were 4 cases of BHS and ES simultaneously present during the same period (see Fig. 2 for details). The types of ES include GTCS in 3 cases, myoclonic in 1 case, focal seizures in 4 cases, epileptic spasms in 2 cases, tonic in 1 case, and atypical absence seizures in 1 case; 2 cases had 2 or more types of ES (VEEG, cEEG, disease course records and follow-up provided).

### 3.1. VEEG results (see Table 1)

Two cases had normal background activity, 5 cases (71.42%) had abnormalities (slowed background rhythm, burst inhibition, or high dysrhythms), and 5 cases (71.42%) had interictal epileptiform discharges (multifocal or generalized); Ictal EEG: The BHS and ES of 7 cases were confirmed by VEEG. There were 8 “slow” patterns and 5 “slow-flat-slow” patterns in all 13 BHS of 7 cases. During the occurrence of BHS, 5 cases had slowed background activity, and 4 cases had IED. The types of ES: 2 cases had anoxic tonic-clonic seizures (ATCS), 1 case had epileptic spasms, 1 case had myoclonic, 2 cases had focal seizures or status epilepticus, 1 case had occiputal spikes and febrile seizures.

**Table 1:** The VEEG results of 7 pediatric patients.

Case	VEEG age	Background activity	IED	Seizure manifestation	Ictal EEG
1	2 yr	Slowed	Occiputal spike, spike-slow wave	Crying → holding breath, cyanosis, slowed heart rate, head tilted back, loss consciousness	Slow-flat-slow patterns, 50 s
2	10 mo	Normal	No	Crying → holding breath, cyanosis, slowed heart rate, head tilted back → Binocular gazed, bilateral limbs tonic → limbs clonic	Slow patterns → generalized voltage reduction → generalized spike wave rhythm, with gradually increasing amplitude and decreasing frequency, lasting for about 105 s
3	4 mo	Numerous δ activity(occipital)	Multifocal sharp (occipital)	Crying → holding breath, cyanosis, 2 times	Slow patterns, 30–45 s
	8 mo	Hypsarrhythmia	Numerous multifocal sharp and spike (occipital)	Nodding with upper limb adduction, chain, 15–105 times/chain	Generalized high amplitude slow wave complex low amplitude fast rhythm or HFO, lasting for 1–1.5 s, accompanied by synchronized rhombic EMG burst of the deltoid muscle (see Fig. 3)
4	7 mo	Diffused β activities	Central and temporal sharp	Quick blinking, limb or body shaking	Wide spread spike-slow wave secondary to central lobe, accompanied by synchronous upper limb EMG burst of about 50 ms
				Crying → holding breath, cyanosis, 4 times	Slow patterns, 30–50 s
5	10 d	Burst-suppression	Multifocal spikes	Gazing, lip cyanosis, and limbs clonus	Rhythmic discharge and evolution starting from multiple foci
	7 mo	Slowed	No	Crying → holding breath, cyanosis, slowed heart rate, head tilted back, loss consciousness 3 times	Slow patterns. 1 times or slow-flat-slow patterns. 2 times 30–50 s.
6	29 d	Delayed maturity, no awakening – sleep cycle	Multifocal spikes	Binocular deviation, with or without left or right limb clonus	Rhythmic discharge originating from the right or left brain area and evolving sustained state
	8 mo	Slowed	A few of multifocal spikes	Crying → holding breath, cyanosis, slowed heart rate, head tilted back, loss consciousness	Slow-flat-slow patterns. 35 s (see Fig. 4)
7	1 yr 5 mo	Normal	No	Crying → holding breath, cyanosis, slowed heart rate, head tilted back → Binocular gazed, bilateral limbs tonic → limbs clonic	Slow patterns → generalized voltage reduction → voltage and frequency of generalized spike wave rhythm were evolved over time, lasting for about 130 s (see Fig. 5)

EEG = electroencephalogram, IED = interictal epileptiform discharge, VEEG = video electroencephalogram.

**Figure 3.**
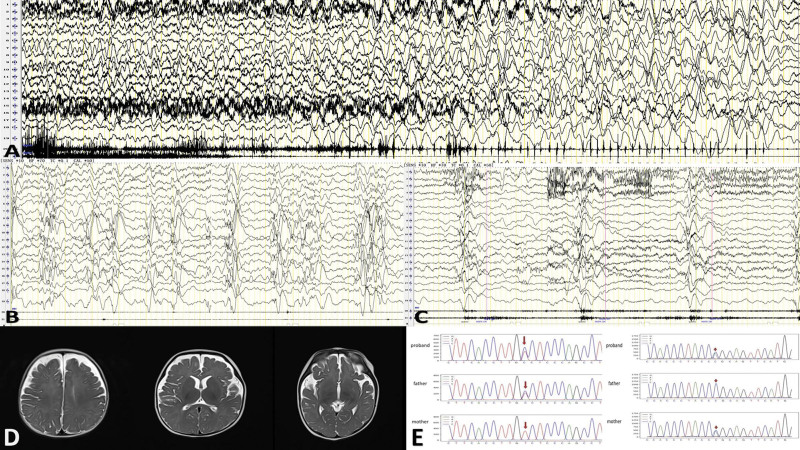
Case 3, male. (A) the “slow” patterns with BHS at 4 mo age. (B and C) VEEG at 8 mo age: B showed hypothalamia (significant at the occiput); (C) 3 times spasms. (D) MRI showed Bilateral front and temporary extra brain spaces slightly increased, (E) LCT gene c.4583A > G, c.4867-5C > A heterozygous mutation. BHS = breath-holding spells, MRI = magnetic resonance imaging, VEEG = video electroencephalogram.

**Figure 4.**
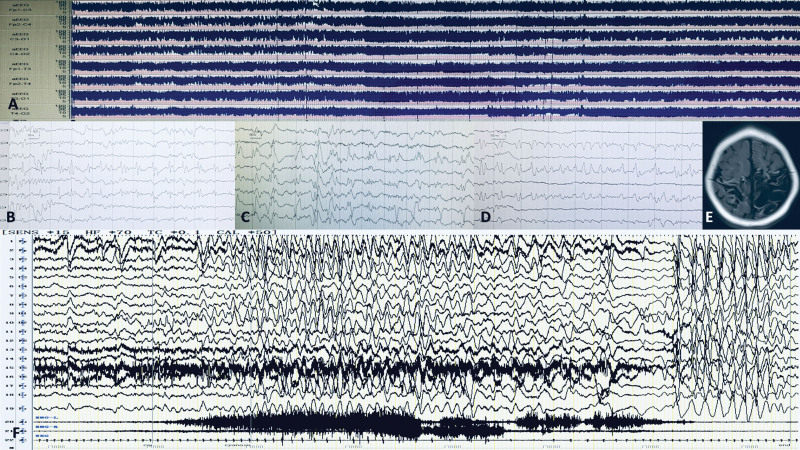
Case 6 female. (A to D) Bedside cEEG at 29 d age: (A) aEEG showed sustained state of focal seizures (with a large amount of arched elevation at the lower boundary). (B, C, and D) 3 times focal seizures respectively: EEG showed rhythmic discharge starting from the left or right brain area. (E) head MRI showed cephalomalacia. (F) VEEG at 1 yr 5 mo age: slow-flat-low patterns with BHS. aEEG = amplitude e integrated EEG, BHS = breath-holding spells, cEEG = continuous EEG, MRI = magnetic resonance imaging, VEEG = video electroencephalogram.

**Figure 5.**
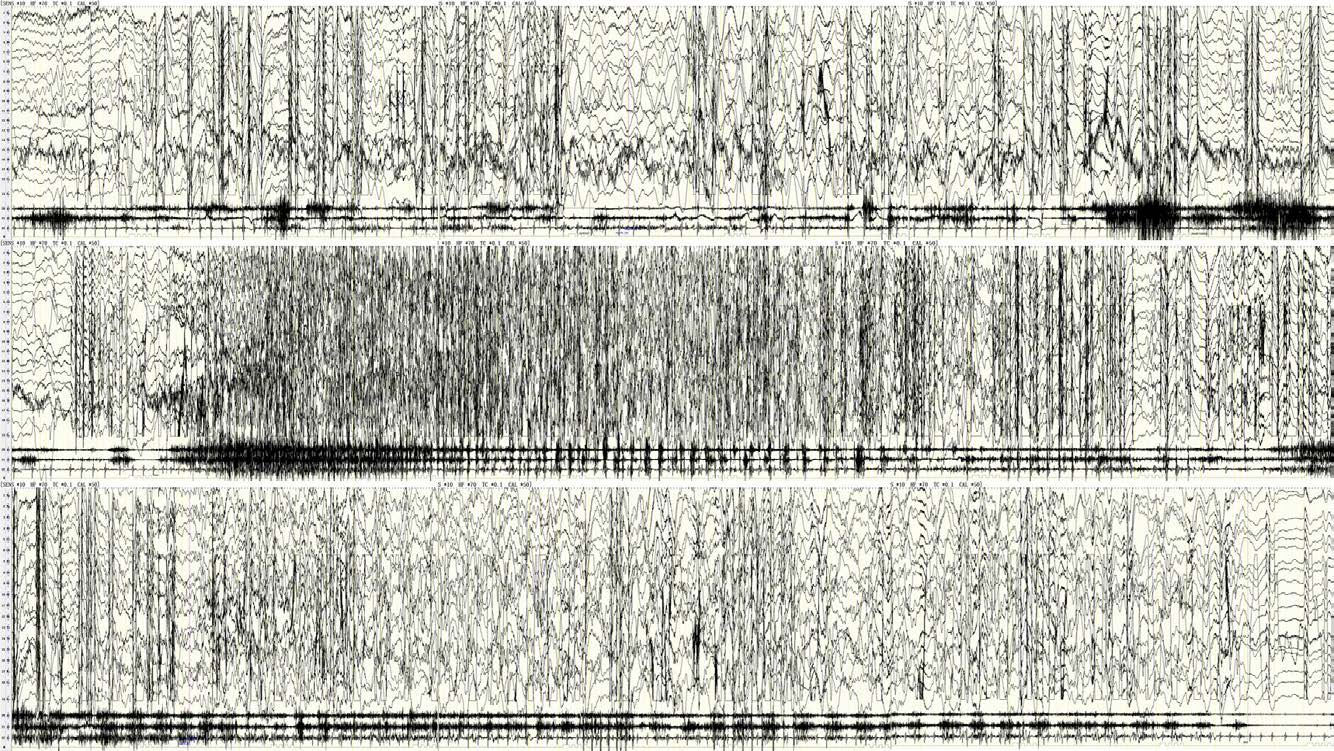
Case 7, male, 1 yr 5 mo. A complete process of GTCS caused by BHS: “slow” patterns → generalized voltage reduction → voltage and frequency of generalized spike wave rhythm evolved over time, lasting for about 130 s. BHS = breath-holding spells, GTCS = generalized tonic clonic seizures.

### 3.2. Other laboratory results

Five cases of abnormal head MRI (71.42%): 2 cases of cerebral softening and atrophy, 2 cases of widening of bilateral frontal or/and temporal extra brain spaces, 1 case of patchy high signal shadow, 4 cases (57.14%) of patent foramen ovale (PFO) on cardiac ultrasound. Only one guardian of a child with epilepsy and encephalopathy was willing to undergo genetic testing, as he detected the LCT gene mutation. Six cases (85.71%) had varying degrees of developmental and behavioral abnormalities: four cases had severe developmental delay (all with severe EEG or/and MRI abnormalities), one case had mild developmental delay with learning difficulties, and one case had hyperactivity. Family history: 7 cases had no history of BHS in their family, and 2 cases had a history of epilepsy. Past history: one case of febrile convulsion, one case of bilirubin encephalopathy, one case of herpes simplex virus encephalitis and larngomalacia, one case of severe HIE complicated with purulent meningitis, and one case of neonatal encephalopathy and larngomalacia.

### 3.3. Treatment and follow-up

In BHS, 7 cases of BHS were treated with psychological and behavioral therapy to reduce stimulation and avoid crying. Five cases stopped before the age of 3 years, one case (1 year and 9 months) had occasional attacks, and one case (1 year and 6 months) had frequent attacks 1 to 2 times per day. In ES, 2 cases with ATCS were not treated with antiseizure medication. Seizures were free after BHS was stopped. One case of focal seizures in the neonatal period and one case of persistent focal seizures in early infancy was free after etiology and anticonvulsant treatment, one case with febrile convulsions pre BHS was free by self, one case of myoclonic and epileptic spasms during the course of the disease was reduced after VPA and VGB treatment. One case (Case 3) presented with multiple types of seizures during the course of the disease, and multiple antiseizure medications and ketogenic diet were administered, resulting in uncontrolled. The detailed clinical data of 7 cases are summarized in Table 2.

**Table 2:** The detailed clinical dates BHS of 7 pediatric patients.

Case	Gender	Age of BHS	Head MRI	Cardiac ultrasound	Age of ES onset/type	Family/Past history	ASMs	Developmental-behavioral	Follow-up time/outcomes
1	Male	1 yr 11 mo–2 yr 6 mo	Normal	Normal	8 mo /GTCS by fever	Grandma and father history of convulsions /no	No	Mild developmental delay, Learning disability	6 yr 6 mo /EEG lost follow-up, Seizure-free
2	Male	8 mo–1 yr 6 mo	Normal	Normal	10 mo /ATCS	No /no	No	Hyperactivity	4 yr 7 mo /Seizure-free
3	Male	4 mo–2 yr	Bilateral frontal and temporal extra brain spaces slightly increased	PFO	8 mo /spasms, Focal seizures, tonic, atypical absence	No /Congenital larngomalacia, LCT gene mutations	ACTH TPM VGB VPA LTG KD	Severe developmental delay	3 yr 2 mo /ES uncontrolled 1–2 times/d
4	Male	7 mo–2 yr	Bilateral diffuse encephalomalacia and encephalatrophy	PFO	7 mo /myoclonic, spasms	No /neonatal Severe HIE and purulent meningitis	VPA VGB	Severe developmental delay	3 yr 4 mo /a few ES
5	Male	7 mo–now	Bilateral temporal extra brain spaces slightly increased	PFO	10 d /focal seizures	No /Neonatal bilirubin encephalopathy	No	Severe developmental delay	9 mo /BHS uncontrolled 1–2 times/d
6	Female	10 mo–now	Bilateral diffuse encephalomalacia/Cystic degeneration	PFO	1 mo /focal seizures SE	No /Neonatal herpes simplex encephalitis, larngomalacia	LEV	Severe developmental delay	5 mo /EEG slowed and spikes, occasional BHS
7	Male	5 mo–1 yr 8 mo	Patchy and slightly longer T2 signal shadows in the white matter	Normal	1 yr 5 mo /ATCS	Mother has depression, father has epilepsy /no	No	Normal	4 mo /Seizure-free

ACTH = adrenocorticotropic hormone, ASMs = antiseizure medications, ATCS = anoxic tonic-clonic seizures, BHS = breath-holding spells, ES = epileptic seizures, HIE = hypoxic-ischemic encephalopathy, LEV = levetiracetam, LTG = lamotrigine, KD, ketogenic diet, MRI = magnetic resonance imaging, PFO = patent foramen ovale, TPM = topiramate, VPA = valproate, VGB = Vigabatrin.

Statistical analysis results for the BHS-only and the BHS + epilepsy groups: the onset age of breath-holding attacks in the BHS-only group was earlier than that in the BHS + epilepsy groups, and the 2 groups had statistical significance (*P* < .05). The abnormal rates of head MRI and EEG in the BHS-only group were lower than those in the BHS + epilepsy groups, and the difference between the 2 groups was statistically significant (*P* < .05). The BHS-only group had normal interictal EEG, while 5 out of 7 patients in the BHS + epilepsy group had epileptic waves in their interictal EEG. The incidence of the BHS + epilepsy group combined with other diseases was higher than that of BHS-only group, and the difference between the 2 was statistically significant (*P* < .05). The rate of developmental delay in the BHS + epilepsy group is higher than that in the BHS-only group, and there is a statistical difference between the 2 groups (*P* < .05) (see Table 3).

**Table 3:** Comparison of the patient demographics, developmental status, comorbid conditions, and auxiliary examination results between BHS-only and BHS + epilepsy groups.

	Onset age (mo)	Relief age of BHS (mo)	Gender (male: female)	EEG (normal: unmormal)	MRI (normal: unmormal)	Comorbid (yes: no)	Developmental status (normal: delay)
BHS-only	10 (8, 14)	18 (14, 25)	23:6	29:0	1:28	4:25	27:2
BHS + epilepsy	7 (5, 8)	18 (14, 20)	5:1	3:4	2:5	4:3	2:5
Statistical value	*Z* = 1.989	*Z* = 0.12	χ^2^ = 0.148	χ^2^ = 18.643	χ^2^ = 18.643	χ^2^ = 6.131	χ^2^ = 14.992
*P*	.047	.904	.692	.000	.000	.021	.000

BHS = breath-holding spells, EEG = electroencephalogram, MRI = magnetic resonance imaging.

## 4. Discussion

Comorbidities are common among people with epilepsy and are associated with poorer clinical outcomes and quality of life, greater use of health resources, and increased expenditure. Becoming aware of the associated mechanisms and their uncertainty is central to understanding the relationships between epilepsy and comorbid health conditions, which have implications for diagnosis and screening, medical management.^[8]^ Depression, anxiety, and other mental illnesses are more common in children with epilepsy of school age or above, and developmental delay is more common in infancy.^[9]^ Infancy is the foundational stage of adaptability, gross motor skills, personal and social, language and fine motor skills development. Both BHS and ES may cause damage to the developing midbrain. Therefore, the co-occurrence of BHS with ES deserves our high attention.

BHS is a reflex TAS, the ictal EEG being stereotyped “slow” pattern or “slow-flat-slow” patterns. This is due to sudden brief respiratory pauses and reduced cardiac output leading to cerebral hypoxia and circulatory disorders. All BHS of 7 cases were confirmed by VEEG. BHS with obvious tonic spasms needs to be distinguished from GTCS, while BHS with obvious cyanosis or pallor without unclear motor symptoms should be distinguished from focal autonomic seizures, especially in children with abnormal discharge and developmental delay. This ictal EEG of GTCS is a generalized spike wave rhythm with onset and evolution. Focal autonomic seizures in infants can also manifest lip cyanosis, slowed heart rate, and impaired consciousness. Ictal EEG is often a rhythmic discharge originating from the temporal lobe and evolving.^[10]^ Vlachou et al^[11]^ described a 12-month-old girl with benign epilepsy caused by a PRRT2 gene mutation in an infant. She experienced recurrent seizures since she was 9 months old, with initial symptoms of pale face and occasional purple lips, similar to reflexive anoxic seizures. As a result, the seizure symptoms can interfere with our judgment. VEEG is the gold standard for diagnosing BHS and ES. ES may occur in the acute phase of various severe encephalopathies in the neonatal period, but neonatal ES often lacks reliable clinical symptoms and can only be monitored by VEEG.^[12]^ amplitude integrated EEG or combined with original EEG are recommended for bedside cEEG monitoring in newborns.

PFO is an embryonic remnant of the fetal circulation, present in 20 to 25% of adults. The role of a PFO in exacerbating hypoxemic medical conditions remains less understood.^[13]^ The mechanism by which BHS can cause breathing difficulties and pauses is a brief spasm of the trachea during exhalation due to sudden crying, which is not related to PFO. Caydiac syncope is caused by a sudden decrease in cardiac out, resulting in pale or cyanotic complexion and unclear consciousness. Therefore, BHS should exclude Long Q-T Syndrome and other heart diseases, especially in pallid, frequent spells occurring at a younger age and having a positive family history.^[14]^ Congenital Airway Anomalies include a variety of conditions that cause respiratory distress in neonates and infants. The most frequent congenital airway anomalies is laryngomalacia.^[15]^

There is little literature on the BHS comorbidity with ES, mostly case reports. Khan et al^[16]^ describe an 11-year-old boy with Bainbridge–Ropers syndrome who has daily intractable seizures reported since birth, developmental delay, autistic features, and feeding difficulties. He has frequent episodes of breath-holding accompanied by dystonic posturing with right leg extension and head turning without ictal EEG correlating. Among our cases, 2 had BHS leading to GTCS. Ranza et al^[17]^ reported 2 cases and showed the association of de novo SCN8A variants and anoxic-ES. One of them had focal or generalized seizures and autonomic symptoms triggered by orthostatism; the second had BHS triggered by pain or exercise leading to tonic-clonic seizures. The mechanism of anoxic-ES may be that hypoxia in brain tissue caused by hypoxemia triggers abnormal electrical activity of neurons, leading to ES.

The exact pathophysiology of BHS is not yet clear, but dysregulation of the autonomic nervous system seems to play a role. The etiology of ES includes genetics, structure, metabolism, infection, immunity, etc, and these etiologies can overlap. In this paper, we included structural abnormalities caused by infection and severe HIE, one case of severe bilirubin encephalopathy caused by metabolic abnormalities, and the other 4 cases that may be related to genetics, but only one case (case 3) was willing to undergo genetic testing, because he had multiple types of ES difficult to control, which had transformed from infantile epileptic spasms syndrome to Lennox–Gastaut syndrome. Unfortunately, CNV, chromosome karyotype, and mitochondrial genes are all normal. The second-generation sequencing showed heterozygous mutations in the LCT gene c.4583A > G, and c.4867-5C > A. The LCT gene is a gene that regulates the expression of lactase, and the mutated phenotype is mainly lactose intolerance.^[18]^ There is currently no evidence to confirm its association with epileptic encephalopathy.

The treatment of BHS generally does not require medication and focuses on psychological and behavioral therapy, as well as removing triggers. BHS has age-dependent characteristics, with a significant decrease or cessation of attacks after the age of 3. In severe or repeated cases, they expose the brain to hypoxia and cause a lot of stress in parents. In these cases, the clinician should consider therapy. Belladonna and risperidone (after ruling out seizure disorders) were highly effective to alleviate severe BHS in young children, without any major adverse effects.^[19,20]^ BHS is highly frequent and complicated by prolonged syncope, convulsions and even status epilepticus. In these cases, response to medical treatment is often unsatisfactory. Pacemaker implantation is a possible therapeutic option.^[21]^ In our cases, one case of BHS still had frequent attacks, as the current age was 1 year and 6 months. Despite the frequency of attacks, the severity of the attacks has decreased as age increases. Therefore, his parents had not yet agreed to take more proactive treatment measures. The treatment principle of ES was to target the etiology, seizure type, and epilepsy syndrome for precise treatment. Although the attribute of ATCS is ES, the seizures were caused by TAS, and the treatment principle was to remove BHS.^[22]^

The correlation between BHS and ES: generally speaking, BHS does not cause permanent neuronal damage,^[23]^ but frequent and prolonged holding of breath may lead to neuronal metabolic dysfunction or damage.^[24]^ Is the incidence of ES higher in these children with BHS due to possible hypoxic brain injury? In a survey of 519 BHS cases, 6 children (1.16%) had a pathological EEG, 4 (7.71%) of which had concomitant epilepsy.^[25]^ Similar to the prevalence of active epilepsy in the normal population at 6.38%.^[26]^ Our study showed that in children with coexisting BHS and ES, the age of stoped in most BHS was similar to those without ES. The ES of acute phase were seizures-free after etiology treatment and anticonvulsant therapy, while infantile epileptic spasms syndrome^[27]^ and Lennox–Gastaut syndrome are led by genetic and structural factors, which are inherently difficult to treat. Treatment difficulties for spasms and tonics are related to the etiology and epilepsy syndrome. In summary, the treatment and prognosis of coexisting BHS and ES, whether they exist simultaneously or sequentially, must follow their respective disease patterns. Some studies suggest that the delayed maturation of brainstem myelin sheath formation may play a role in the etiology of BHS in children.^[28]^ Therefore, children with brain injuries such as epilepsy and developmental delay may be more susceptible to BHS when provoked. BHS and ES may have a dual impact on the baby’s brain and may exacerbate each other. More in-depth and detailed basic and clinical research is needed.

## 5. Conclusion

BHS generally has a good prognosis and does not require excessive treatment, but for some patients with abnormal head MRI, EEG, or developmental delay, secondary ES may occur and require antiseizure medication. Accurate diagnosis of BHS and ES was a prerequisite for rational treatment and care in these infants; BHS and ES had their own unique EEG patterns, and VEEG was the gold standard for diagnosis and differentiation. Both BHS and ES comorbidities may cause damage to the developing brain during infancy and should be taken seriously. Given the limited number of cases, this paper is only “throw away a brick in order to get a gem.” We hope that there will be more exciting research on this topic in the future.

## Acknowledgments

We wish to thank the patients for participating in this study.

## Author contributions

**Conceptualization:** Zeshu Ning.

**Data curation:** Bo Chen, Xiaojun Kuang.

**Formal analysis:** Zeshu Ning.

**Investigation:** Bo Chen, Hongjun Fang.

**Methodology:** Bo Chen, Hongjun Fang.

**Resources:** Lijun Wang.

**Writing – original draft:** Bo Chen, Xiaojun Kuang.

**Writing – review & editing:** Bo Chen, Hongjun Fang, Xiaojun Kuang.
